# Immediate Effects of Dynamic and Rigid Taping on Static and Dynamic Plantar Pressure Distribution in Individuals with Hallux Valgus: A Cross-Over Study

**DOI:** 10.3390/jcm15155985

**Published:** 2026-08-01

**Authors:** Berrak Varhan, Beyza Tanrıöğen, Hüseyin Melih Göktuğ Akpulat, Hatice Karabulut

**Affiliations:** 1 Department of Physiotherapy and Rehabilitation, Faculty of Health Sciences, Istinye University, 34010 Istanbul, Turkey; beyza.tanriogen@istinye.edu.tr (B.T.); hatice.karabulut@istinye.edu.tr (H.K.); 2 Department of Physiotherapy and Rehabilitation, Institute of Graduate Studies, Istinye University, 34396 Istanbul, Turkey; hmelih.akpulat@igdir.edu.tr; 3 Department of Therapy and Rehabilitation, Vocational School of Health Services, Iğdır University, 76000 Iğdır, Turkey

**Keywords:** hallux valgus, dynamic taping, rigid taping, plantar pressure, foot deformity

## Abstract

**Background/Objectives**: We aimed to investigate the effects of different taping techniques commonly used in the management of hallux valgus (HV) on plantar pressure distribution under static and dynamic conditions. **Methods**: Thirty participants, including 15 individuals with HV and 15 healthy controls, were included. Individuals with mild–moderate HV were identified using the Manchester scale, while healthy participants were determined using the Foot Posture Index. Plantar pressure distribution was assessed using the Freemed Maxi pressure platform (Sensor Medica, Italy). Participants were first evaluated without taping. Rigid taping and dynamic taping with mechanical correction techniques were then applied in a randomized order with a 24-h interval. Participants rested for 30 min after each taping application to evaluate the immediate effect. The primary outcome variables were static total pressure, forefoot load, rearfoot load, and dynamic forefoot, rearfoot, medial, and lateral plantar loads measured using pedobarography. **Results**: Data were analyzed using a repeated-measures General Linear Model in SPSS 21.0. No significant differences were observed between groups in static total pressure, forefoot load, or rearfoot load (*p* = 0.965, *p* = 0.928, and *p* = 0.829, respectively). Significant effects were found for dynamic forefoot and rearfoot loads (*p* = 0.036 for both variables). Dynamic medial and lateral loads showed no significant differences (*p* = 0.338). **Conclusions:** Dynamic taping modified dynamic plantar loading during gait but did not restore plantar pressure patterns comparable to those of healthy individuals.

## 1. Introduction

Hallux valgus (HV) is one of the most common chronic foot deformities and represents the most prevalent pathological condition affecting the great toe [1,2]. HV is a progressive deformity characterized by lateral deviation of the great toe at the first metatarsophalangeal (MTP) joint, and pronation and medial deviation of the first metatarsal bone, leading to alterations in the anatomical structure and biomechanical function of the foot [3,4]. If the deformity is allowed to progress without treatment, foot function, daily activities, and health-related quality of life may be negatively affected. HV is associated with symptoms such as pain, balance impairments, walking difficulties, and problems related to footwear selection [5].

The etiology of HV remains partially unclear [1]. Since HV develops gradually over time, it has been suggested that cumulative trauma and repetitive loading may contribute to its development [6]. Ligamentous laxity has also been proposed as a contributing factor, as it may exacerbate instability at the first tarsometatarsal and metatarsophalangeal joints, thereby facilitating the progression of HV [7].

One of the important steps in the management of foot pathologies is the objective analysis of foot mechanics under dynamic conditions. Hallux valgus is a common foot deformity that can alter plantar pressure distribution [8]. Theoretically, due to its structural alterations, HV may modify plantar pressure distribution and load-bearing capacity in the forefoot [9]. Typically, a reduction in loading beneath the hallux occurs, accompanied by compensatory increases in pressure in other regions of the foot, which may contribute to secondary foot problems such as metatarsalgia [10]. Previous studies have reported reduced load-bearing capacity of the hallux and first metatarsal region during the mid-stance and push-off phases of gait, resulting in compensatory increases in loading beneath the central metatarsal heads [11].

Various conservative approaches have been proposed for the management of HV, including exercise [3,12], orthoses (such as toe separators, night splints, and anti-pronation insoles) [13], taping techniques, and manual or manipulative therapy. Among these approaches, rigid and dynamic taping are frequently used in clinical practice. Previous studies have reported that both taping methods may reduce pain, improve function, and help prevent deformity progression [14,15,16].

Dynamic taping is a therapeutic method that aims to produce mechanical effects on the musculoskeletal system through the application of elastic adhesive tapes [17]. The proposed mechanism of action of dynamic taping is related to both mechanical support and neuromuscular stimulation. Mechanically, dynamic taping provides elastic support while allowing the preservation of joint range of motion without completely restricting movement. Furthermore, following application, the tape may generate recoil forces on the underlying tissues, potentially contributing to improved stability in the targeted region. From a neuromuscular perspective, the tension generated by the tape may enhance sensory input and thereby improve proprioceptive awareness.

Rigid taping, on the other hand, is a non-elastic adhesive taping method commonly used in athletes to prevent injuries or reduce the risk of injury [18]. This method is often applied to reduce pain, provide proprioceptive feedback during activity, and limit excessive joint motion. Compared with dynamic tapes, rigid tapes exhibit greater stiffness and limited elasticity, thereby providing stronger mechanical support and more pronounced restriction of joint motion in the applied region.

Although several studies have evaluated the effects of dynamic taping [14,19] and rigid taping [16,20,21] on hallux valgus, the number and scope of these studies remain limited. Existing research has mainly focused on clinical outcomes such as pain and functional improvement, and studies directly comparing these taping techniques using objective biomechanical measurements reflecting foot loading are lacking.

Although several studies have investigated plantar pressure distribution in individuals with HV [11,22], no study has examined the immediate effects of dynamic and rigid taping techniques on plantar pressure distribution while directly comparing these two approaches. Therefore, investigating the effects of these taping techniques on plantar pressure distribution may contribute to a better understanding of the mechanical role of taping interventions in the management of hallux valgus.

Although rigid and dynamic taping have different theoretical mechanisms of action, both techniques are commonly used in the conservative management of hallux valgus with the aim of improving foot mechanics and reducing abnormal loading. Therefore, comparing their immediate biomechanical effects may contribute to clinical decision-making regarding taping selection.

Based on the current evidence, the aim of this study was to analyze the effects of dynamic and rigid taping techniques on plantar pressure distribution in individuals with hallux valgus.

## 2. Materials and Methods

### 2.1. Study Design

**Figure 1:**
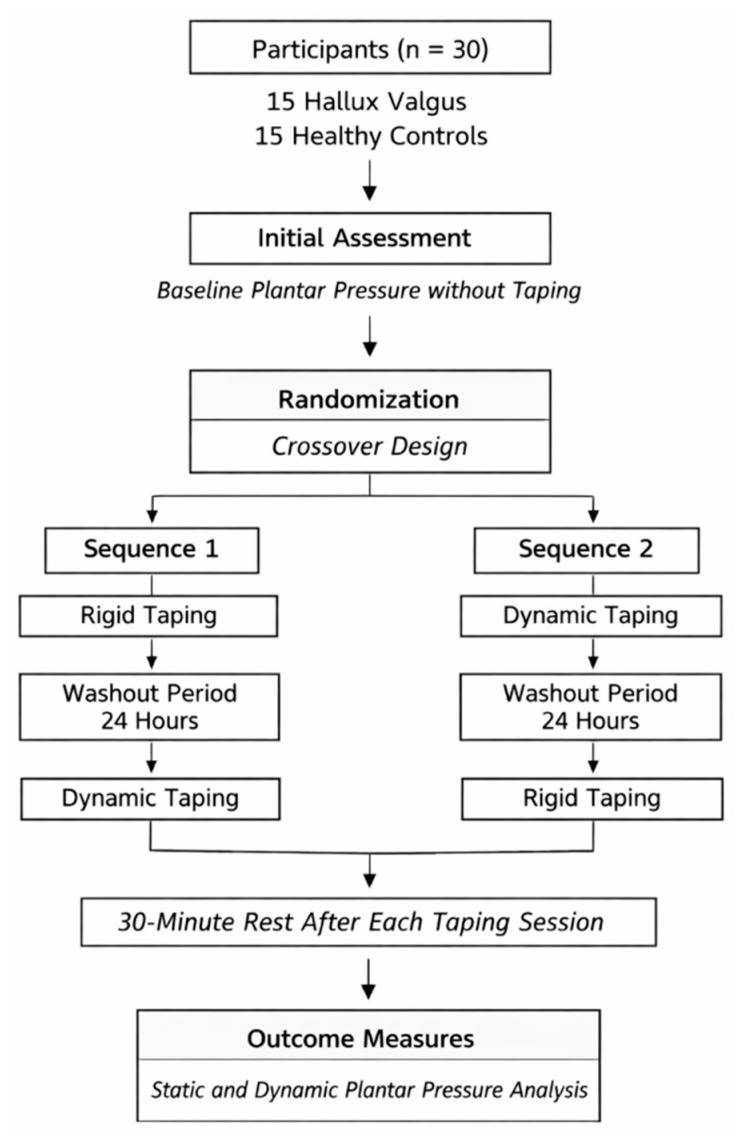
Study Flowchart.

This study employed a mixed design. Individuals with hallux valgus underwent a randomized crossover intervention (rigid taping and dynamic taping), whereas healthy controls were evaluated only once and served as a reference group. The study was conducted at the Istinye University Physiotherapy and Rehabilitation Application and Research Center. Ethical approval was obtained from the Istinye University Human Research Ethics Committee in 2022 (Protocol No.: 22/77). Participants were recruited between May and November 2022. All individuals were informed about the study procedures and provided written informed consent before participation. The study flowchart is shown in Figure 1.

The diagnosis of hallux valgus (HV) was established by an orthopedic and traumatology specialist based on clinical evaluation, with particular consideration given to the alignment of the first metatarsophalangeal joint.

### 2.2. Participants

A total of 30 individuals, including 15 participants diagnosed with hallux valgus and 15 healthy controls, were included in the study. A simple randomization procedure using sealed opaque envelopes was employed. Rigid taping and dynamic taping interventions were applied to the individuals with HV at specified intervals, whereas healthy participants were evaluated as the control group. The sample size was calculated using G*Power software (version 3.1.9.7). Based on the study by Karabicak et al. (2025) [15], which examined the acute effects of taping in individuals with hallux valgus, the effect size was set as f = 0.30 (moderate–large effect). With an alpha level of 0.05 and a statistical power of 0.80, the required minimum total sample size was calculated to be 24 participants. To compensate for possible data loss and to increase statistical power, 30 participants (15 HV patients and 15 healthy controls) were included, resulting in an estimated statistical power of approximately 0.88. The effect size was derived from the study by Karabicak et al. because it was the most methodologically comparable study evaluating immediate effects of taping interventions in hallux valgus patients.

Participants were included if they had no orthopedic disability, were between 30 and 55 years of age, and had a non-rigid hallux valgus deformity. Individuals with hallux valgus were required to present a deformity classified as grade 2 or higher according to the Manchester scale or a hallux valgus angle greater than 15°. Participants were excluded if they had a history of lower extremity surgery, systemic diseases such as rheumatoid arthritis, neurological disorders, or any skin lesions or allergies that could interfere with the application of taping.

### 2.3. Outcome Measures

#### 2.3.1. Sociodemographic Data

After signing the informed consent form, participants completed a form that collected sociodemographic data including sex, age, body weight, height and body mass index (BMI).

#### 2.3.2. Manchester Scale

The Manchester scale is a valid and reliable method used to determine the severity of hallux valgus deformity. The scale consists of standardized images representing normal foot alignment (A) and mild (B), moderate (C), and severe (D) hallux valgus deformities. Participants were evaluated using this scale, and individuals corresponding to images B, C, or D were included in the study [23,24].

#### 2.3.3. Foot and Ankle Assessments

*Hallux Valgus Angle*

The angle between the long axes of the proximal phalanx and the first metatarsal was measured from the dorsal aspect of the foot using a goniometer and recorded in degrees under both weight-bearing and non-weight-bearing conditions [25].

*Assessment of Medial Longitudinal Arch Height*

The height of the medial longitudinal arch (MLA) was evaluated using the navicular drop test.

*Navicular Drop Test*

The navicular drop test was used to assess the degree of foot pronation during weight-bearing. The participant was first seated, and the distance between the navicular tuberosity and the floor was measured. The measurement was repeated when the participant stood and bore weight on the foot. The difference between the two measurements indicated the navicular drop value (mm).

*Metatarsal Width Measurement*

The widest distance between the medial and lateral concavities at the metatarsal level while the foot was in contact with the ground was measured using a caliper and recorded under both weight-bearing and non-weight-bearing conditions.

*Subtalar Joint Angle*

While the participant was in the prone position, the angle between the calcaneus and the distal one-third of the lower leg was measured using a goniometer (valgus: −, varus: +). The measurement was repeated when the participant stood and bore full weight on the foot.

*Foot Posture Index*

The Foot Posture Index was used to assess deviations in foot posture. Reference points specified in the test protocol were identified by palpation and scored between −12 and +10. Scores indicate whether the foot is positioned in varus or valgus alignment. Feet scoring between 0 and +5 are considered normal, and only individuals within this range were included in the study [26].

*Plantar Pressure Assessment*

Plantar pressure distribution was measured using a pedobarography system, specifically the freeMed Maxi platform (Sensor Medica, Roma, Italy). Before the measurements, participants’ body weight and BMI were recorded. During testing, participants stood barefoot on the platform with feet parallel, arms resting alongside the body, and gaze directed forward. Participants maintained this orthostatic position for 5 s. Measurements were recorded at a sampling frequency of 50 Hz, and the following parameters were analyzed:

- Total plantar pressure (%);
- Forefoot pressure (%);
- Rearfoot pressure (%);
- Dynamic medial pressure (%);
- Dynamic lateral pressure (%).

**Figure 2:**
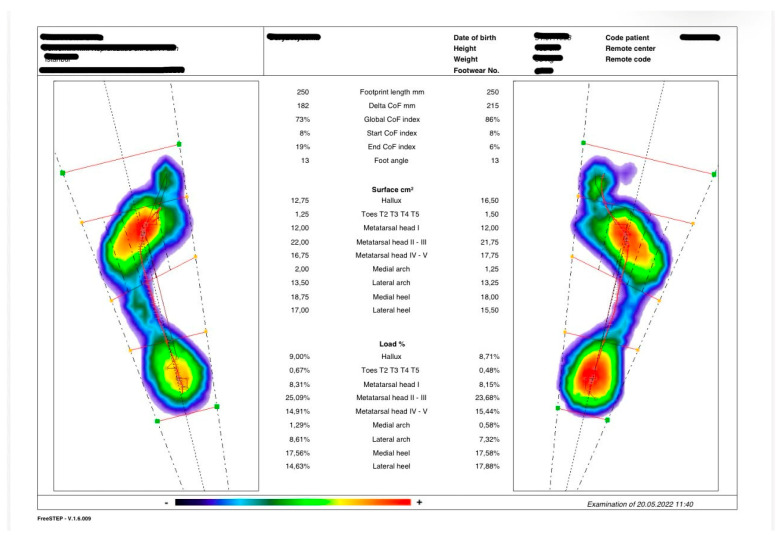
Dynamic Analyses. Color scale represents pressure intensity (blue: low, red: high). The center of force trajectory is marked with a dashed line.

**Figure 3:**
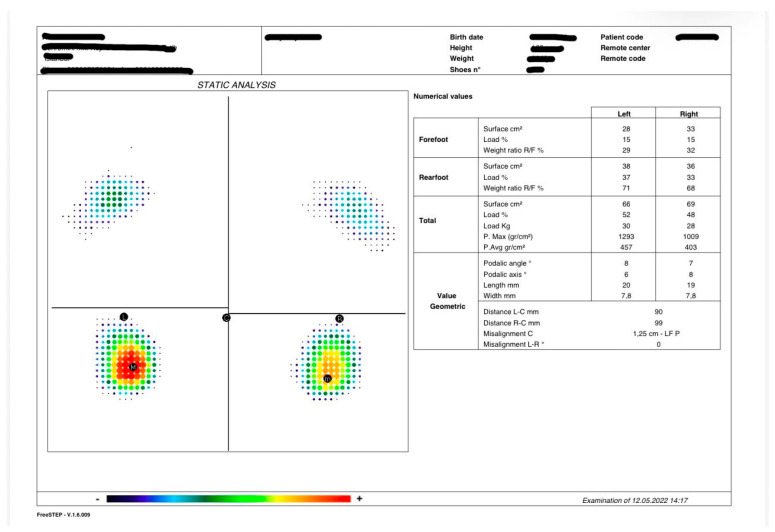
Static Analyses. The color scale represents pressure intensity (blue: low, red: high). The table presents: Forefoot/Rearfoot—regional surface area, load percentage, and weight distribution ratio; Total—total surface area, load, and maximum/average pressure (gr/cm^2^); Podalic angle/axis—the angular and axial orientation of the foot; Length/Width—footprint dimensions; Distance L-C/R-C—distance of the foot center from the midline; Misalignment C—lateral shift of the center of gravity; Misalignment L-R—angular asymmetry between the right and left foot.

Representative examples of the static and dynamic plantar pressure analyses are shown in Figure 2 and Figure 3.

### 2.4. Intervention Procedures

The study included 15 individuals diagnosed with hallux valgus and 15 healthy controls. Individuals with HV received rigid taping and dynamic taping interventions.

For plantar pressure assessment, each participant was first evaluated without taping while walking on the platform. Subsequently, rigid taping was applied, and participants waited 30 min to allow the effect of the tape to occur [27,28]. A 30-min period was allowed after tape application based on previous studies suggesting that the mechanical and proprioceptive effects of taping may require a short accommodation period. Participants were then asked to walk on the platform, and the measurements were recorded. To ensure that the effect of the tape had completely dissipated, there was a 24-h interval before the next intervention. Because the study investigated immediate effects of taping, a 24-h washout period was considered sufficient to minimize any residual mechanical or sensory influence of the previous intervention [29]. On the following day, dynamic taping was applied, participants again waited 30 min, and plantar pressure measurements were repeated.

#### 2.4.1. Dynamic Taping Application

**Figure 4:**
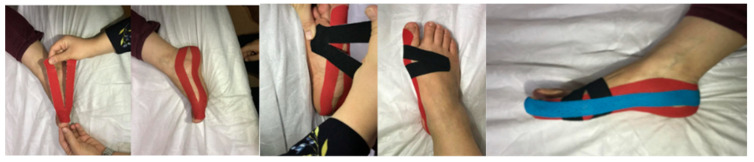
Steps of Dynamic Taping Protocol.

Dynamic taping was applied to correct hallux valgus deformity using two Y-strips and one I-strip using a mechanical correction technique. The aim of the taping was to guide the tissue and joint toward a neutral position. Dynamic taping was applied using Kinesio Tex^®^ tape (Kinesio Holding Corporation, Albuquerque, NM, USA), the taping procedure is shown in Figure 4).

The taping protocol was applied in three stages according to previously described procedures in the literature [15,19,30].

#### 2.4.2. Rigid Taping Application

**Figure 5:**
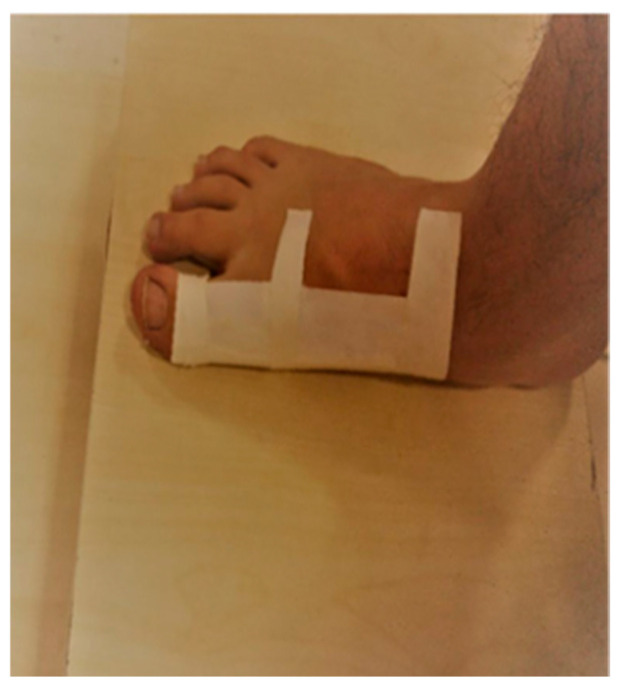
Rigid Taping.

Participants were positioned in long sitting position, with the feet relaxed and in a neutral position. The great toe was gently pulled laterally, and circular taping was applied around the toe and along the medial aspect of the foot. Subsequently, the toe was abducted and additional taping was applied toward the medial side of the foot. Finally, circular taping was repeated around the great toe and the medial midfoot to increase the traction effect [31] (the taping procedure is shown in Figure 5).

### 2.5. Statistical Analysis

Statistical analyses were performed using IBM SPSS Statistics (version 26; IBM Corp., Armonk, NY, USA). The normality of the data distribution was evaluated using the Shapiro–Wilk test. Descriptive statistics were presented as mean ± standard deviation for continuous variables and frequency (percentage) for categorical variables.

The primary analysis was conducted in the hallux valgus (HV) group using a within-subject design. To evaluate the effects of taping condition (baseline, rigid taping, and dynamic taping) on plantar pressure variables, a one-way repeated-measures analysis of variance (RM-ANOVA) was performed. When a significant main effect was detected, Bonferroni-adjusted pairwise comparisons were conducted to identify differences between conditions. Partial eta-squared (η^2^p) values were calculated to determine effect sizes.

Healthy control participants were not included in the repeated-measures model because they only underwent a single assessment and did not participate in the crossover intervention. Therefore, the control group was used as an external reference group. Secondary analyses were performed to compare healthy controls with each HV condition (baseline, rigid taping, and dynamic taping) using independent-samples *t*-tests for normally distributed variables or Mann–Whitney U tests when normality assumptions were violated. Cohen’s d effect sizes were calculated for between-group comparisons.

Demographic characteristics were compared between the HV and healthy control groups using independent-sample *t*-tests for continuous variables and chi-square tests for categorical variables. Statistical significance was set at *p* < 0.05 for all analyses.

## 3. Results

Three conditions were included in the study: rigid taping, kinesio taping, and control. Individuals with hallux valgus underwent a randomized crossover intervention where the rigid taping and kinesio taping conditions were applied to the same group of participants (*n* = 15). Therefore, demographic characteristics were identical for both taping conditions. The mean age of the participants in the taping group was 28.73 ± 13.07 years, the mean body weight was 65.93 ± 12.97 kg, the mean height was 1.697 ± 0.069 m, the and mean body mass index (BMI) was 22.77 ± 3.49 kg/m^2^. In the control group (*n* = 15), the mean age was 28.87 ± 12.91 years, the mean body weight was 66.30 ± 12.15 kg, the mean height was 1.705 ± 0.08 m, and the mean BMI was 22.23 ± 3.50 kg/m^2^. Regarding sex distribution, there were 12 females and 3 males in the taping group and 11 females and 4 males in the control group. No statistically significant differences were observed between groups in terms of demographic variables (*p* > 0.05) (Table 1).

### 3.1. Static Plantar Pressure Parameters

Repeated-measures ANOVA revealed no significant effect of taping condition on static plantar pressure parameters. Forefoot load (F(2,28) = 0.11, *p* = 0.896, η^2^p = 0.008), rearfoot load (F(2,28) = 0.30, *p* = 0.741, η^2^p = 0.021), and total pressure (F(2,28) = 0.04, *p* = 0.957, η^2^p = 0.003) remained unchanged across baseline, rigid taping, and dynamic taping conditions. These findings indicate that neither taping application produced an immediate effect on static plantar pressure distribution (Table 2).

### 3.2. Dynamic Plantar Pressure Parameters

Significant effects of taping condition were observed for dynamic plantar loading. Dynamic forefoot load demonstrated a significant reduction across conditions (F(2,28) = 4.62, *p* = 0.018, η^2^p = 0.248), whereas dynamic rearfoot load significantly increased (F(2,28) = 4.62, *p* = 0.018, η^2^p = 0.248). The effect sizes were moderate to large. Bonferroni-adjusted pairwise comparisons revealed that dynamic forefoot load was significantly lower following dynamic taping compared with baseline measurements (mean difference = 4.00, 95% CI = 0.31–7.69, *p* = 0.032). Similarly, dynamic rearfoot load was significantly greater following dynamic taping than at baseline (mean difference = 4.00, 95% CI = 0.31–7.69, *p* = 0.032). No significant differences were observed between rigid taping and baseline conditions or between rigid and dynamic taping conditions (all *p* > 0.05) (Table 3).

### 3.3. Comparison with Healthy Reference

Secondary analyses were performed to compare plantar pressure variables between healthy controls and each hallux valgus condition. No significant differences were observed between healthy controls and baseline hallux valgus measurements for static forefoot load, rearfoot load, total pressure, dynamic forefoot load, or dynamic rearfoot load (all *p* > 0.05). Similarly, no between-group differences were detected for static plantar pressure variables following rigid or dynamic taping (all *p* > 0.05). However, dynamic forefoot load and dynamic rearfoot load differed significantly between healthy controls and the rigid taping condition (*p* = 0.037 for both variables) and between healthy controls and the dynamic taping condition (*p* = 0.002 for both variables). Effect size analysis revealed large effects for rigid taping (Cohen’s d = 0.80) and very large effects for dynamic taping (Cohen’s d = 1.24). These findings indicate that the significant changes observed following taping did not result in plantar loading patterns that were closer to those observed in healthy individuals (Table 4).

## 4. Discussion

In the present study, the effects of rigid and dynamic taping applications on plantar pressure distribution in individuals with hallux valgus were investigated. The findings indicated that neither taping method produced an immediate and statistically significant effect on static plantar pressure distribution. Under dynamic conditions, however, limited changes were observed, particularly in forefoot and rearfoot loads. Although dynamic taping showed a tendency to produce loading patterns closer to those observed in healthy individuals for certain parameters, this effect did not reach a clinically meaningful level.

It should be acknowledged that rigid and dynamic taping represent distinct therapeutic approaches. Rigid taping primarily aims to mechanically stabilize and restrict joint motion, whereas dynamic taping is intended to provide elastic assistance while influencing proprioceptive and neuromuscular control. Therefore, the present comparison should not be interpreted as a comparison of equivalent interventions, but rather as an exploration of how two commonly used taping strategies influence plantar pressure distribution.

Dynamic taping may influence plantar load distribution by assisting hallux alignment and modifying weight transfer during gait. The reduction observed in dynamic forefoot loading together with the increase in rearfoot loading may indicate a redistribution of plantar forces during the terminal stance and push-off phases. From a biomechanical perspective, such changes may reduce mechanical stress on the first metatarsophalangeal joint and alter the loading strategy adopted during walking.

The absence of significant effects in static plantar pressure measurements may suggest that taping primarily influences dynamic motor control rather than passive standing alignment. Walking requires continuous neuromuscular adaptations and sensorimotor adjustments, whereas quiet standing is a relatively stable task with lower mechanical demands. Therefore, the mechanical support and proprioceptive input provided by taping may become more evident during dynamic activities than during static postures.

Despite inconsistencies in the literature regarding plantar pressure distribution in individuals with hallux valgus, several studies have reported a transfer of load from the painful medial region toward the central and lateral forefoot areas [32]. Dynamic taping has been reported as an effective approach in HV rehabilitation for reducing pain and edema, improving proprioception, and providing joint support [33]. Short-term studies have also demonstrated improvements in the hallux valgus angle (HVA), as well as gait stability and balance parameters following dynamic taping interventions [33]. In our study, dynamic taping brought the mean values of dynamic medial and lateral loads closer to those measured in healthy individuals, suggesting a potential short-term influence on gait and balance parameters. However, most studies on dynamic taping have primarily focused on therapeutic and proprioceptive effects on muscles and joints [14,19], highlighting a gap regarding its influence on motor control mechanisms in individuals with HV. Furthermore, the limited number of studies examining dynamic [14,19] and rigid taping [16,20,21] in HV have generally focused on linear foot posture measurements rather than regional load distribution during standing and gait.

Clinical studies indicate that feet with hallux valgus typically exhibit reduced plantar pressure beneath the hallux, with negative correlations between plantar pressure values and hallux valgus angles [9]. Hutton et al. compared plantar pressures of 65 individuals with HV and 64 healthy participants using a force-transducer plate and reported higher pressures beneath the third to fifth metatarsal heads [22]. In contrast, Mickle et al. observed significantly higher peak pressures beneath the second metatarsal head [34]. Similarly, Yamamoto et al. reported increased forefoot pressure during walking in feet affected by hallux valgus, particularly beneath the lesser metatarsal heads [35]. These inconsistencies may be explained by differences in HV severity, medial soft tissue alterations associated with bunion formation, hyperkeratosis beneath the second and third metatarsal heads, variations in connective tissue tension around the metatarsal heads, and progressive valgus deviation of the first metatarsal.

Another possible explanation for heterogeneous findings is methodological variation between plantar pressure measurement systems. Many studies have used single pedobarographic plates or in-shoe pressure measurement systems [36]. Pedobarographic plates provide high spatial resolution but may restrict natural gait due to their limited measurement area. In contrast, in-shoe systems allow for prolonged activity monitoring but typically have fewer sensors and lower spatial resolution. In the present study, plantar pressure measurements were obtained using the FreeMed Maxi (Sensor Medica) pedobarography system, enabling objective evaluation of both static and dynamic parameters and representing a practical option for clinical and field-based assessments [36].

Previous studies examining taping interventions in HV have reported similar findings. Jeon et al. reported that a four-week rigid taping intervention reduced the hallux valgus angle from 21.95° to 18.73° [14], suggesting that angular correction may contribute to more balanced plantar pressure distribution. In our study, dynamic taping appeared to be more effective than rigid taping in modifying dynamic plantar pressure distribution. Similarly, Żłobiński et al. reported that dynamic taping immediately reduced the hallux valgus angle, improved rearfoot alignment, and normalized foot load distribution [33]. These findings suggest that dynamic taping may contribute to normalization of plantar pressure patterns by modifying load transfer within the foot.

Galica et al., analyzing 6393 feet, reported reduced loading beneath the hallux and a shift of load toward regions outside the hallux during gait in individuals with HV compared with a reference group [10]. In contrast to our findings, no significant difference was observed in medial forefoot loading in their study. Nyska et al. reported lower pressure values in the rearfoot and heel regions in individuals with HV during standing and walking [11]. In the present study, no change was observed in static rearfoot loading, whereas an increase in dynamic rearfoot loading was detected, suggesting that taping applications may influence plantar load distribution.

Martínez-Nova et al. reported that increased dynamic pressure in individuals with HV was mainly localized in the forefoot, particularly at the metatarsal heads, with no significant differences in rearfoot pressure [37]. In contrast, our findings demonstrated increased dynamic rearfoot loading, which may indicate that rigid and kinesio taping increase load transfer toward the rearfoot during the terminal stance and push-off phases of gait.

Other studies provide additional context. Yokozuka et al. reported decreased plantar pressure beneath the second to fifth toes and second to fourth metatarsal heads, with increased lateral foot pressure in individuals with HV [38]. In our study, lateral loading values in the kinesio taping group approached those observed in healthy individuals, suggesting a potential normalization of lateral load distribution. Similarly, Taş et al. reported comparable static pressure distributions in asymptomatic individuals with mild HV and healthy participants [39]. In a similar vein, Sarika reported differences in plantar pressure distribution and gait parameters between athletes with and without hallux valgus, further supporting the influence of HV on load distribution during dynamic activities [40]. Consistent with this finding, rigid and kinesio taping in our study did not produce statistically significant changes in static loading; however, certain parameters approached the values observed in healthy individuals.

Although statistically significant changes were observed in dynamic forefoot and rearfoot loading, their clinical relevance remains uncertain. The magnitude of the observed changes was relatively small, and no patient-reported outcomes such as pain, comfort, or functional status were evaluated. Therefore, caution is warranted when interpreting these findings from a clinical perspective.

Several limitations should be considered. First, the sample size was relatively small, which may have reduced statistical sensitivity. Second, only immediate effects were examined and therefore, long-term efficacy cannot be determined. Third, healthy controls were not included in the crossover intervention and were used solely as a reference group. Finally, the findings are limited to individuals with mild-to-moderate hallux valgus and may not be generalizable to more severe deformities. Although the sample size met the a priori power requirements, the relatively small number of participants may have limited the ability to detect subtle between-condition differences and increased the risk of Type II errors. Therefore, the findings should be interpreted cautiously and may not be generalizable to all individuals with hallux valgus. Another limitation is that rigid and dynamic taping have different theoretical mechanisms and clinical objectives. Consequently, direct comparison between these interventions should be interpreted cautiously. The present study focused on their immediate biomechanical effects on plantar pressure distribution rather than their overall therapeutic effectiveness.

Contrary to our initial expectation, the changes observed following dynamic taping did not consistently approach the loading characteristics of the healthy reference group. Comparisons with the reference values demonstrated moderate-to-large between-group effect sizes after taping, suggesting that although dynamic taping altered plantar load distribution, these changes should not be interpreted as normalization of plantar loading patterns.

To the best of our knowledge, no previous study has directly compared the effects of dynamic and rigid taping on plantar pressure distribution. Therefore, the findings of this study may contribute a novel perspective to the existing literature.

## 5. Conclusions

Dynamic taping, but not rigid taping, induced significant alterations in dynamic plantar pressure distribution in individuals with hallux valgus, characterized by decreased forefoot loading and increased rearfoot loading during gait. No significant effects were observed for static plantar pressure parameters. Although taping modified plantar loading patterns, these changes did not consistently resemble those observed in healthy individuals. Further studies are needed to determine their clinical relevance and long-term effects.

## Figures and Tables

**Table 1:** Demographic Variables.

Variable	Rigid Taping (*n* = 15)	Dynamic Taping (*n* = 15)	Control (*n* = 15)	*p* Value
Sex (Female/Male)	12/3	12/3	11/4	0.892
Age (years)	28.73 ± 13.07	28.73 ± 13.07	28.87 ± 12.91	*p* > 0.999
Body Weight (kg)	65.93 ± 12.97	65.93 ± 12.97	66.30 ± 12.15	*p* > 0.999
Height (m)	1.697 ± 0.069	1.697 ± 0.069	1.705 ± 0.08	*p* > 0.999
BMI (kg/m^2^)	22.77 ± 3.49	22.77 ± 3.49	22.23 ± 3.50	*p* > 0.999

**Table 2:** Effects of Baseline, Rigid Taping, and Dynamic Taping on Plantar Pressure Variables in Individuals with Hallux Valgus.

Variable	Baseline (Mean ± SD)	Rigid Taping (Mean ± SD)	Dynamic Taping (Mean ± SD)	F (df = 2,28)	*p*	Partial η^2^
Static Forefoot Load (%)	18.47 ± 6.41	19.07 ± 4.85	18.60 ± 5.44	0.11	0.896	0.008
Static Rearfoot Load (%)	30.60 ± 5.41	30.07 ± 5.20	30.73 ± 4.54	0.30	0.741	0.021
Total Pressure (%)	49.07 ± 4.53	49.13 ± 4.63	49.33 ± 4.70	0.04	0.957	0.003
Dynamic Forefoot Load (%)	62.27 ± 4.96	60.67 ± 3.99	58.27 ± 5.06	4.62	0.018 *	0.248
Dynamic Rearfoot Load (%)	37.73 ± 4.96	39.33 ± 3.99	41.73 ± 5.06	4.62	0.018 *	0.248

* Statistically significant at *p* < 0.05.

**Table 3:** Bonferroni-Adjusted Pairwise Comparisons for Significant Dynamic Plantar Pressure Variables.

Variable	Comparison	Mean Difference (95% CI)	*p*
Dynamic Forefoot Load	Baseline vs. Rigid	1.60 (−1.75 to 4.95)	0.647
Dynamic Forefoot Load	Baseline vs. Dynamic	4.00 (0.31 to 7.69)	0.032 *
Dynamic Forefoot Load	Rigid vs. Dynamic	2.40 (−1.34 to 6.14)	0.309
Dynamic Rearfoot Load	Baseline vs. Rigid	−1.60 (−4.95 to 1.75)	0.647
Dynamic Rearfoot Load	Baseline vs. Dynamic	−4.00 (−7.69 to −0.31)	0.032 *
Dynamic Rearfoot Load	Rigid vs. Dynamic	−2.40 (−6.14 to 1.34)	0.309

* Bonferroni-corrected significance level *p* < 0.05.

**Table 4:** Comparison of Plantar Pressure Parameters Between Healthy Controls and Hallux Valgus Conditions.

Variable	Healthy Controls	HV Baseline	*p*	d	HV Rigid Taping	*p*	d	HV Dynamic Taping	*p*	d
Forefoot Load (%)	22.20 ± 7.18	18.47 ± 6.41	0.144	0.55	19.07 ± 4.85	0.172	0.51	18.60 ± 5.44	0.133	0.57
Rearfoot Load (%)	28.00 ± 6.75	30.60 ± 5.41	0.254	0.42	30.07 ± 5.20	0.356	0.34	30.73 ± 4.54	0.204	0.47
Total Pressure (%)	50.20 ± 4.33	49.07 ± 4.53	0.489	0.26	49.13 ± 4.63	0.520	0.24	49.33 ± 4.70	0.604	0.19
Dynamic Forefoot Load (%)	63.73 ± 3.65	62.27 ± 4.96	0.365	0.34	60.67 ± 3.99	0.037 *	0.80	58.27 ± 5.06	0.002 **	1.24
Dynamic Rearfoot Load (%)	36.27 ± 3.65	37.73 ± 4.96	0.365	0.34	39.33 ± 3.99	0.037 *	0.80	41.73 ± 5.06	0.002 **	1.24

* *p* < 0.05, ** *p* < 0.01. Cohen’s d: 0.2—small; 0.5—medium; 0.8—large effect.

## Data Availability

The data presented in this study are available from the corresponding author upon reasonable request. The data are not publicly available due to privacy and ethical restrictions.

## Abbreviations

The following abbreviations are used in this manuscript:

BMI	Body Mass Index
GLM	General Linear Model
HV	Hallux Valgus
HVA	Hallux Valgus Angle
MLA	Medial Longitudinal Arch
MTP	Metatarsophalangeal
SPSS	Statistical Package for the Social Sciences
